# Effect of Pretreatment on the Corrosion Behavior of AHSS CP 780 Analyzed by Electrochemical Techniques

**DOI:** 10.3390/ma19020225

**Published:** 2026-01-06

**Authors:** Citlalli Gaona-Tiburcio, Demetrio Nieves-Mendoza, Jesus Manuel Jaquez-Muñoz, Jose Cabral-Miramontes, Erick Maldonado-Bandala, Brenda Baltazar-Garcia, Miguel Angel Baltazar-Zamora, Francisco Estupinan-Lopez, María Lara-Banda, Javier Olguin-Coca, Juan Pablo Flores-De los Rios, Facundo Almeraya-Calderon

**Affiliations:** 1 Universidad Autónoma de Nuevo León, FIME, Centro de Investigación e Innovación en Ingeniería Aeronáutica (CIIIA), San Nicolás de los Garza 66455, Mexico; jose.cabralmr@uanl.edu.mx (J.C.-M.); francisco.estupinanlp@uanl.edu.mx (F.E.-L.); maria.laraba@uanl.edu.mx (M.L.-B.); facundo.almerayacld@uanl.edu.mx (F.A.-C.); 2 Facultad de Ingeniería Civil, Universidad Veracruzana, Xalapa 91000, Mexico; erimaldonado@uv.mx (E.M.-B.); pao.baltazar.08@gmail.com (B.B.-G.); mbaltazar@uv.mx (M.A.B.-Z.); 3 Centro de Ciencias de la Ingeniera, Universidad Autonóma de Aguascalientes, Aguascalientes 20340, Mexico; jesus.jaquez@edu.uaa.mx; 4 Área Académica de Ingeniería y Arquitectura, Universidad Autónoma del Estado de Hidalgo, Carretera Pachuca-Tulancingo Km. 4.5, Hidalgo 42082, Mexico; olguinc@uaeh.edu.mx; 5 Tecnológico Nacional de Mexico-Instituto Tecnológico de Chihuahua, Av. Tecnologico 2909, Chihuahua 31130, Mexico; jpfloresr@itch.edu.mx

**Keywords:** E-coat, electrochemistry, corrosion, pretreatment, corrosion, AHSSs

## Abstract

To reduce CO_2_ emissions into the environment, the automotive sector uses microalloyed structural steels coated with electrophoretic paint in various components, such as the chassis, to reduce weight and increase corrosion resistance. AHSSs are coated with electrophoretic paint (E-coat). Still, to improve adhesion, they undergo a pretreatment, such as zinc phosphate or zirconium oxide. This research will analyze the effects and behavior of these coatings during corrosion on a complex-phase (CP) 780 AHSS using different electrochemical techniques, including cyclic potentiodynamic polarization (CPP), electrochemical noise (EN), and electrochemical impedance spectroscopy (EIS). The CP 780 AHSS was immersed in a 3.5 wt. % sodium chloride solution. Results show that AHSS CP 780 presented a mixed corrosion mechanism due to the heterogeneity of the surface of the zinc phosphate and zirconium oxide pretreatments. On the other hand, the samples with an E-coat paint coating and pretreatment (Zn_3_(PO_4_)_2_/E-coat and ZrO_2_/E-coat) have the lowest current densities with values of 6.44 × 10^−11^ 1.02 × 10^−9^ A/cm^2^ and also do not show a tendency towards localized corrosion or negative hysteresis.

## 1. Introduction

The use of special steels has allowed the automotive sector to increase vehicle efficiency by reducing fuel consumption and, consequently, CO_2_ emissions [1,2]. Various components in automobile chassis manufacturing are made from advanced high-strength steels (AHSSs) such as CP (complex-phase), depending on their microstructure [1,2,3,4,5]. CP steels exhibit good toughness (high energy absorption) due to delayed recrystallization, which allows the formation of small grains with a fine, mixed microstructure. This microstructure contains retained fractions of martensite (γ′), austenite (γ), and pearlite (ferrite + iron carbide) in a ferrite/bainite matrix. Their properties are also often improved when microalloying elements such as niobium or titanium form carbides or nitrides. Complex-phase (CP) steels, with their fine microstructure, exhibit high tensile strength (YS) and elongation under tension, similar to that of dual-phase (DP) steels. Furthermore, their wear and fatigue resistance can be improved through heat treatment [6,7,8,9].

Corrosion management and the application of innovative technologies in the automotive industry have enabled manufacturers to achieve a balance among design, materials, and processing. Controlling corrosion through advanced paint/coating technology and the use of corrosion-resistant materials translates into competitive advantages in the automotive sector [10,11].

To increase adhesion between the epoxy coating (paint) and the substrate and improve corrosion resistance, numerous studies in the literature have addressed conversion coatings (pretreatments), such as chromates and phosphates, as efficient and cost-effective alternatives [12,13,14,15,16]. The pretreatment of a metal surface with phosphoric acid (H_3_PO_4_) results in a coating composed primarily of iron phosphate and phosphates of some other heavy metals (aluminum, zinc, cadmium, and tin). Phosphate conversion coatings, particularly zinc phosphate (Zn_3_(PO_4_)_2_), are commonly used in the metalworking industry due to their porous film. When the substrate surface is in contact with phosphoric acid, a chemical reaction occurs between the acid and the substrate (e.g., steel) that locally reduces the hydronium cations H_3_O^+^, increasing the pH and causing the precipitation of the dissolved salt onto the surface [16,17,18,19].

Studies on corrosion in automotive components (e.g., chassis) have identified problems where AHSSs tend to exhibit localized corrosion in specific areas. CP780 steel has been reported to be susceptible to hydrogen-induced corrosion or galvanic corrosion when exposed to environments containing sodium chloride (NaCl), causing a galvanic couple [20,21,22,23,24,25]. Some conversion pretreatments, such as zinc phosphates, induce galvanic corrosion; therefore, selecting the correct coating is important [26]. In the automotive sector, zinc coatings are widely used on high-alloy steels (AHSSs), even though these pretreatments initiate a galvanic corrosion reaction in the material, making it susceptible to localized corrosion [27].

Different conventional electrochemical techniques such as linear polarization resistance (LPR), potentiodynamic polarization (PP), and electrochemical impedance spectroscopy (EIS) have been implemented to determine the corrosion and kinetic mechanisms of the reactions. However, these techniques can alter the electrochemical system with external signals in electrochemical measurements. The use of the electrochemical noise technique for the investigation and monitoring of corrosion has enabled many advances in recent years that are of interest to corrosion science. A particular advantage of electrochemical noise measurements is the possibility to detect and analyze the localized corrosion. Electrochemical noise refers to spontaneous, low-level potential and current fluctuations that occur during an electrochemical process. These transients manifest in potential and current noise that can be exploited in a corrosion map. Transients are linked to anodic and cathodic reactions due to stochastic processes (rupture and re-passivation of the passive or oxide film) and deterministic processes (formation and propagation of pitting) [28,29,30].

Other authors [30,31,32,33,34] improved the LI to determine the type of corrosion using skewness and kurtosis. The statistical values of Equation (1) provide details about the causes and dynamics of corrosion. In 1999, Cottis and Turgoose [35] found a correlation between increased corrosion rate and increased standard deviation and variance.

(1) $$R_{n}=\frac{\sigma_{v}}{\sigma_{I}}*A$$

Valenzuela et al. reported in 2016 that hydrogen is generated on the surface of AHSSs via conversion in the coating’s pore layer, increasing susceptibility to environmental hydrogen embrittlement. These are cathodic reactions in aqueous solution [36,37]. Hydrogen reactions occur due to cathodic reactions (see Equation (2)):

(2) $$O_{2}+{2H}_{2}O+4e^{-}\to 4{OH}^{-}$$

The corrosion problem in AHSS depends on the microstructure (phases) and the type of steel. When retained austenite is present in AHSSs, the corrosion rate is reduced [38,39]. However, authors such as Franceschi et al. [40] have indicated that the amount of bainite increases the corrosion rate, whereas in other cases the conversion coating helps reduce corrosion. Lara et al. and Jabloska et al. [3,41] reported in their studies that advanced high-strength steels exhibit low resistance to pitting corrosion in the presence of electrolytes such as 3.5 wt. % NaCl.

The presence of a two-phase microstructure enables the formation of microgalvanic corrosion cells, leading to localized corrosion in the preferred phases [42]. The martensite transformation increases the corrosion resistance of CP and DP steels. When martensite and bainite are present with ferrite, the ferrite acts as an anode and an active dissolution process occurs [43,44,45].

Electrochemical noise analysis can be performed in time-domain, frequency-domain, time-frequency, and chaotic systems. Authors such as Eden, Cottis, Mansfeld, Turgoose, and Bertocci studied how to relate the type of corrosion to statistical parameters based on data (standard deviation of electrochemical noise in current ($\sigma_{I}$) and potential ($\sigma_{v}$)) from current and potential time series, thus determining the localization index (LI). The Electrochemical noise resistance (Rn) and Linear polarization resistance (LPR or Rp) have a relation, Stern-Geary (Equation (3)) can be applied as an analog relation between them to determine corrosion kinetic. B is a constant with recommended value of 0.026 V for active and 0.052 V for the passive corrosion.

(3) $$i_{corr}=\frac{B}{R_{n}}$$

This work aimed to analyze the effect of pretreatment on the corrosion behavior of AHSS CP 780 steel using electrochemical techniques (cyclic potentiodynamic polarization (CPP), electrochemical noise (EN), and electrochemical impedance spectroscopy (EIS)). The AHSS CP 780 was coated with electrophoretic paint (E-coat), using two pretreatments based on zinc phosphate or zirconium oxide; the samples were evaluated in a 3.5 wt. % sodium chloride solution. AHSSs are used in the automotive industry in environments with heavy snowfall and the use of de-icing salts (NaCl).

## 2. Materials and Methods

### 2.1. Materials

In this investigation, commercially available complex-phase AHSS (CP) was used. The designation CP 780 indicates that it is a complex-phase steel, and 780 indicates its mechanical strength (Rm = 780 MPa). A 10 × 30 cm sheet of CP 780 steel was used. Table 1 reports the chemical composition obtained by atomic absorption spectroscopy (AAS).

The AHSS CP 780 samples underwent two pretreatments before E-coat paint application. The pretreatments were zinc phosphate and zirconium oxide, aimed at improving the adhesion (anchoring) of the electrophoretic paint. The composition of the pretreatments is confidential to the industry.

The zinc phosphate pretreatment consisted of the following stages and parameters: the substrate was degreased in an alkaline medium (Borax) at room temperature, followed by rinsing in water at room temperature, pickling in 10–20% vol. phosphoric acid for 15 min at room temperature, rinsing in water at room temperature, zinc phosphating at a concentration of 8–15% vol. between 60 and 70 °C for 7 min at pH 4, rinsing in water at room temperature, sealing (non-chromic) between 60 and 80 °C for 1 min, and a final rinse in water at 38 °C.

The zirconium oxide pretreatment consisted of the following stages and parameters: the substrate was degreased in an alkaline medium (Borax) at room temperature, followed by two rinses in water with a pH 7 at room temperature, one rinse in deionized water, application of the zirconium oxide coating at a concentration of 4–8% vol. between 25 and 38 °C for 6 min at pH 4, and a rinse in deionized water at room temperature for 2 min.

The electrophoretic coating, E-coat, was applied for 3 min at room temperature, using a voltage of 140 V, and cured in an oven for 20 min at 197 °C.

**Figure 1:**
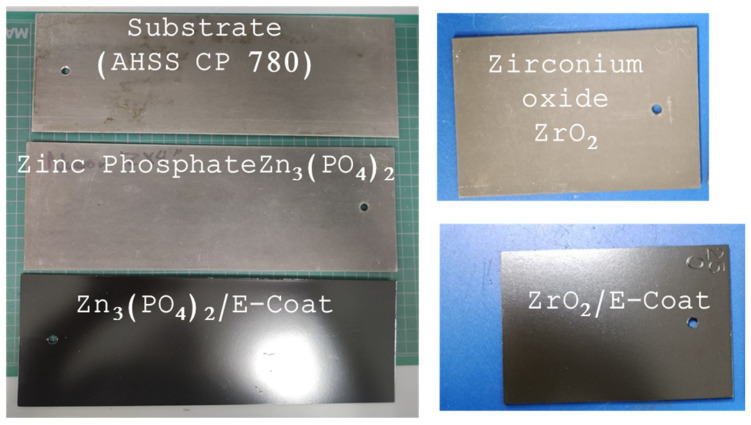
AHSS CP 780 samples with pretreatments.

The samples used in this investigation were the following: AHSS CP 780 (substrate), and AHSS CP 780 with Zn_3_(PO_4_)_2_, ZrO_2_, Zn_3_(PO_4_)_2_/E-coat and ZrO_2_/E-coat (see Figure 1).

### 2.2. Microscopic Characterization

CP 780 steel samples were prepared using metallography [46], employing silicon carbide (SiC) papers of varying grit sizes up to 2500. Alumina (0.1 µm) was used to polish the steel surface. The steel was chemically etched with a 5 wt. % Nital solution. The microstructure and pretreated surface morphology of the samples were examined by scanning electron microscopy (SEM, JEOL-JSM-5610LV, Tokyo, Japan) with a backscattered electron (BSE, JEOL-JSM-5610LV, Tokyo, Japan) detector at 2000× magnification. The chemical composition of the surface elements was determined using energy-dispersive X-ray spectroscopy (EDS, JEOL-JSM-5610LV, Tokyo, Japan). For electrochemical testing, the AHSS CP 780 samples were polished with 800-grit SiC paper.

### 2.3. Electrochemical Corrosion Tests

**Figure 2:**
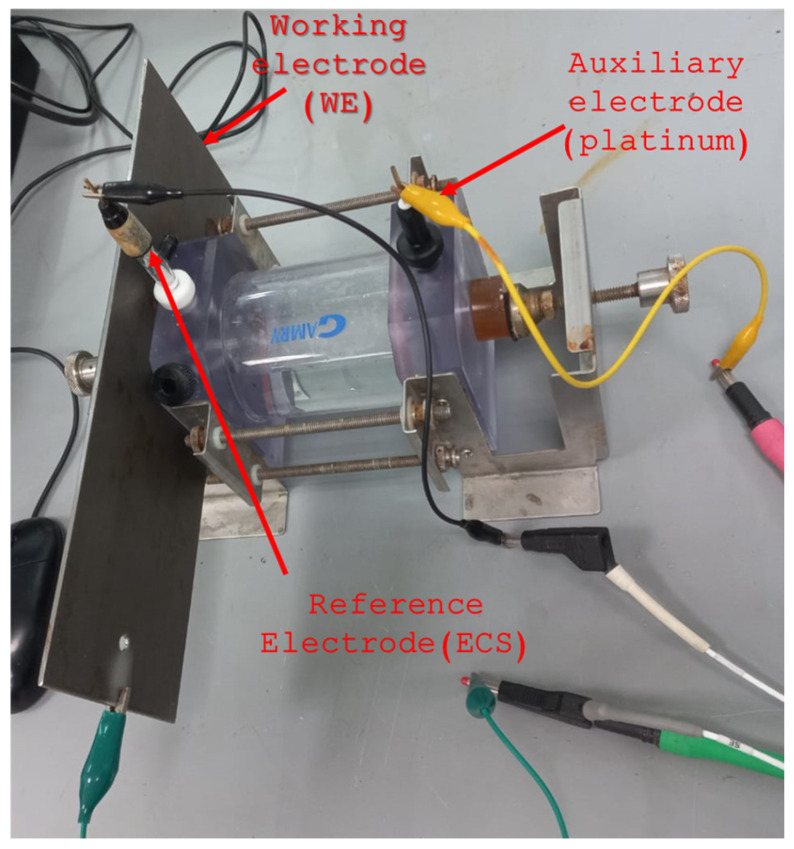
Three-electrode cell for electrochemical corrosion measurements.

The electrochemical corrosion techniques used were cyclic potentiodynamic polarization (CPP), electrochemical noise (EN), and electrochemical impedance spectroscopy (EIS) [47,48]. The electrolyte used was a 3.5 wt. % NaCl solution at room temperature, and duplicate tests were conducted. Electrochemical corrosion measurements were performed using a three-electrode cell (see Figure 2) and a Potentiostat/Galvanostat/ZRA (Solartron 1287A, Bognor Regis, UK).

The electrochemical corrosion of AHSS CP 780 with different pretreatments and paint coatings was studied using cyclic potentiodynamic polarization curves: a potential scan from −0.3 to 1.0 V vs. SCE, starting from the corrosion potential (E_corr_), with a complete polarization cycle at a sweep rate of 0.06 V/min, following the ASTM G61-11 standard [49,50,51]. Analysis of the cathodic and anodic reactions yielded electrochemical parameters such as corrosion potential (E_corr_), anodic–cathodic potential (E_A-C_), corrosion current density (i_corr_), and the cathodic and anodic slopes. Corrosion kinetics were calculated in the 50 mV vs. corrosion potential range in the linear section of the potentiodynamic polarization curves for at least a decade of current, using Tafel extrapolation [52,53,54].

Following ASTM G199-09, EN measurements were performed [55,56]. A scanning speed of 1 data/s was used to collect 1024 data points per trial. A program was developed in MATLAB 2020a (MathWorks, Natick, MA, USA) to analyze information from acquired electrochemical noise data. The time-domain data allows the obtainment of statistical data such as Rn, kurtosis, and skewness. The electrochemical noise (EN) signal, after the DC (DC) component was removed using a ninth-degree polynomial, was analyzed.

In this study, the analysis of kurtosis and skewness was included to determine the type of corrosion because the localization index had limitations, as mentioned in 1995 by Mansfeld and Sun [57], and therefore should be used with caution. However, in the patent of Reid and Eden [58], they indicate that the third and fourth statistical moments, respectively, skewness and kurtosis (see Equations (4) and (5)), can be used to determine the type of corrosion [59], where N is the number of data studied and x is the EN signal.

(4) $$skewness=\;\frac{1}{N}\sum_{i=1}^{N}\frac{{{(x}_{i}-\bar{x})}^{3}}{\sigma^{3}}$$

(5) $$kurtosis=\;\frac{1}{N}\sum_{i=1}^{N}\frac{{{(x}_{i}-\bar{x})}^{4}}{\sigma^{4}}$$

EIS measured a frequency range of 0.01 to 100,000 Hz, with 35 points per decade and a 10 mV RMS amplitude, per ASTM G106-15 [60,61]. The interpretation of Nyquist and Bode plots obtained by EIS is the subject of various theories and models for simulating experimental data. Equivalent circuits provide a physical interpretation that relates each element of the electrical circuit to the electrochemical phenomena occurring at the substrate/pretreatment/paint or pretreatment/electrolyte/paint interface. This results in the determination of the corrosion kinetics of the system under study and allows identification of the corrosion mechanism [62,63,64].

**Figure 3:**
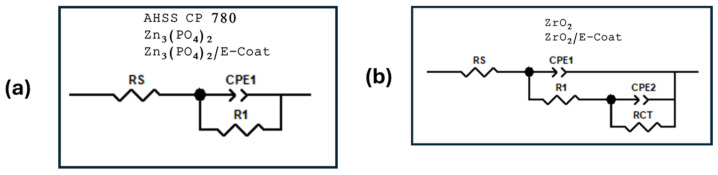
The proposed electrical equivalent circuit (EEC) model. Systems of (**a**) one time-constant and (**b**) two time-constants for CP 780 samples exposed to 3.5 wt. % NaCl solution.

Two equivalent circuits were proposed to model the system’s behavior (see Figure 3a,b). The equivalent circuit models are those that best fit the Nyquist diagrams of the CP780 AHSS samples exposed to a 3.5 wt. % NaCl solution. Rs represents the electrolyte resistance. The constant-phase elements CPE1 and CPE2 represent the oxide layer and protective film elements of the pretreatments, and R1 and R2 represent the resistances of the oxide layer and the zinc phosphate and zirconium oxide layers, respectively. The corrosion process response is given by the electrochemical impedance of the system under study, where the CPEs are represented and often reflect a reactivity distribution [65,66,67]. The simple electrochemical transfer reaction without diffusion can be expressed in terms of a CPE, as in Equation (6):

(6) $$Z(\omega )=\;R_{s}\;+\frac{R_{ct}}{1+{\left(j\omega \right)}^{\alpha }QR_{ct}}$$

Several parameters obtained from the EIS results analysis are Rs, the ohmic resistance; Rct, the charge transfer resistance; and α and Q, frequency-independent parameters (CPEs). If α = 1, Q (capacitance units, μF·cm^−2^) would be the interface capacitance, and if the electrochemical system exhibits behavior attributed to its heterogeneity (α < 1) or to continuously distributed time constants for the charge transfer reactions, the phase angle is associated with the CPE and is frequency-independent (see Equation (7)) [68].

(7) $$Z(\omega )=\;R_{e}\;+\frac{1}{{\left(j\omega \right)}^{\alpha }Q}$$

## 3. Results

### 3.1. Microstructural Characterization (SEM)

**Figure 4:**
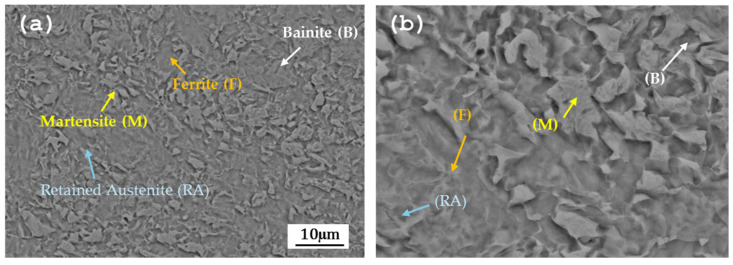
SEM-SE microstructure of AHSS CP780; (**a**) 2000×, (**b**) 5000×.

Figure 4 shows the microstructure of CP780 observed by SEM-BES, where the martensite (M) and retained austenite (RA) phases are observed within a ferrite (F)/bainite (B) matrix.

**Figure 5:**
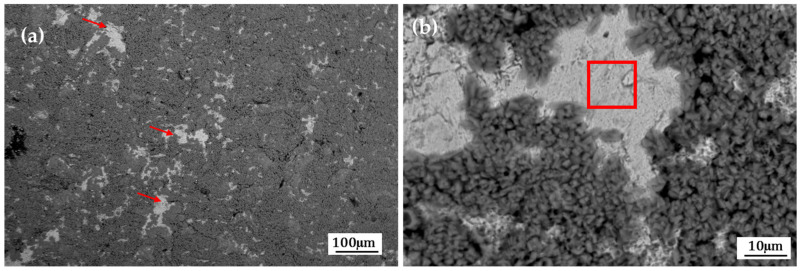
SEM-BSE (**a**,**b**) surface morphology of the sample pretreated with the conversion coating zinc phosphate.

**Figure 6:**
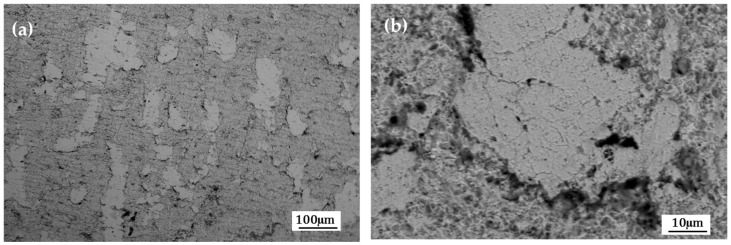
SEM-BSE (**a**,**b**) surface morphology of the sample pretreated with the conversion coating zirconium oxide.

Figure 5 and Figure 6 show the surface morphology obtained by SEM-BES of the samples pretreated with the conversion coatings of zinc phosphate and zirconium oxide. In the morphologies of the samples with pretreatment, areas without coating are observed (Figure 5a and Figure 6a); at higher magnifications, the areas without coating are analyzed (Figure 5b and Figure 6b). In the morphologies of the pretreated samples, uncoated areas are observed (Figure 5a and Figure 6a). At higher magnifications, these uncoated areas are analyzed (Figure 5b and Figure 6b). The coating films are heterogeneous (in the form of clumps or plates) and also exhibit porosity.

### 3.2. Electrochemical Corrosion Measurements

#### 3.2.1. Cyclic Potentiodynamic Polarization

**Figure 7:**
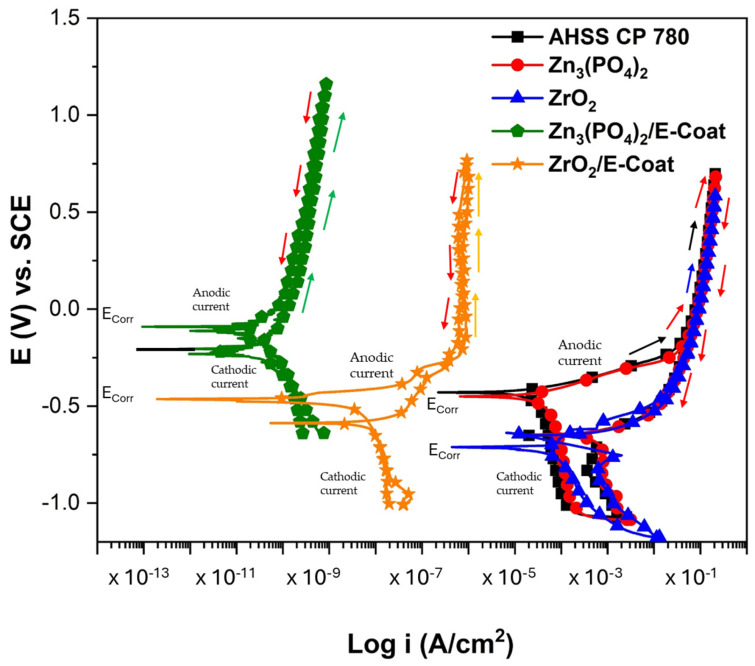
Potentiodynamic polarization curves for AHSS CP 780 exposed to 3.5 wt. % NaCl solution.

Figure 7 shows the potential dynamic polarization of AHSS CP 780, uncoated and with all coating variables. The application of Zn_3_(PO_4_)_2_ on AHSS CP 780 resulted in lower corrosion resistance than AHSS CP 780, generating an inverse effect; the E_corr_ was −0.450 V vs. saturated calomel electrode (SCE), compared to −0.429 V for AHSS CP 780, indicating that the coating has an anodic behavior. Also, i_corr_ presented a value of 3.48 × 10^−5^ A/cm^2^ (see Table 2), meaning an increase in corrosion kinetic; for that reason the % of efficiency of the coating decreases by 44%. The behavior of E_A-C_ indicates that the passive layer generated is stable. The positive hysteresis indicates that the corrosion is a localized process. Something similar occurs with ZrO_2_, where the E_corr_ is anodic (−0.711 V) and the i_corr_ is higher; for that reason, the corrosion kinetics are higher and the coating efficiency is lower (−61%). This indicates that the hysteresis is positive and localized corrosion occurs at the surface.

The behavior of E-coatings is totally different: the Zn_3_(PO_4_)_2_ generated by the E-coat process showed a more noble potential (−0.232 V). The i_corr_ was 6.44 × 10^−11^ A/cm^2^, with an efficiency of 100%, meaning that the corrosion kinetics are lower and the material is protected. However, the E_A-C_ was nobler than E_corr_, with a potential of −0.09 V, indicating that the oxide layer generated is unstable. This sample shows a similar tendency towards passivation. It is important to mention that the hysteresis is negative (as indicated by the arrows on each polarization curve), indicating that the corrosion process changes to uniform corrosion. The ZrO_2_/E-coat also showed good corrosion resistance; the E_corr_ was −0.444 V, and the i_corr_ was 1.02 × 10^−9^ A/cm^2^, with an efficiency near 100%. This is because the corrosion kinetics are significantly reduced. The E_A-C_ was −0.587 V; this active value indicates that the oxide layer generated is more stable. The change in behavior to uniform corrosion is a significant improvement compared with the other samples.

#### 3.2.2. Electrochemical Noise

##### Time-Domain Analysis

**Figure 8:**
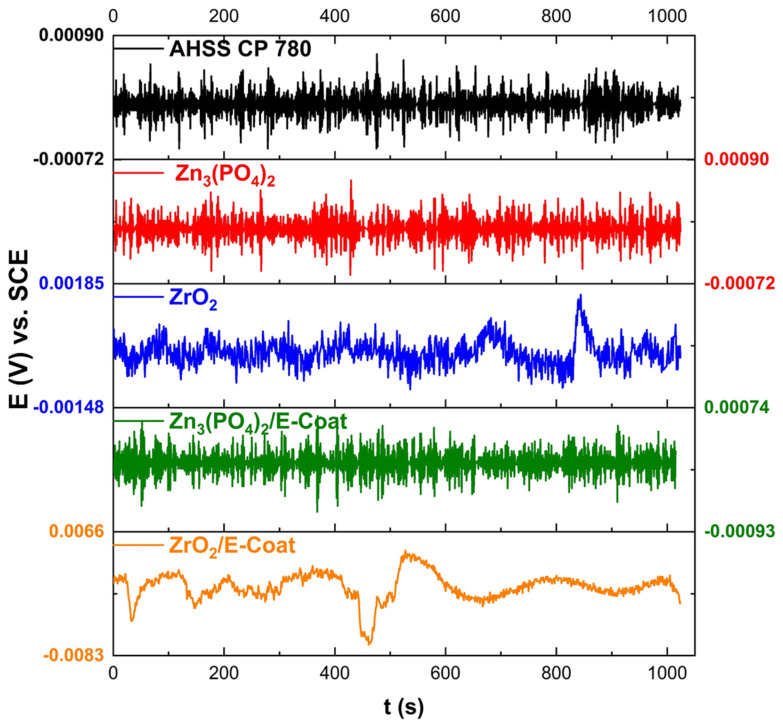
Electrochemical potential noise-time series of samples exposed to 3.5 wt. % NaCl solution.

**Figure 9:**
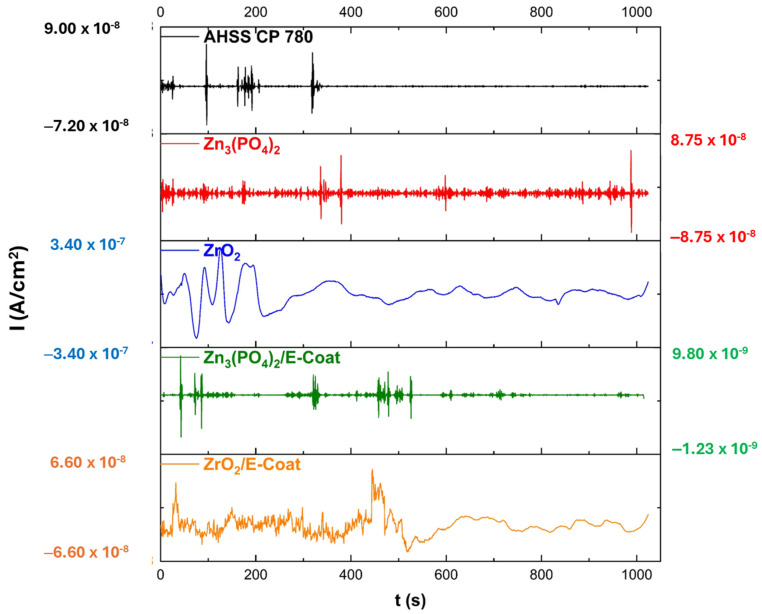
Electrochemical current noise-time series of samples exposed to 3.5 wt. % NaCl solution.

Figure 8 and Figure 9 show the electrochemical potential (EPN) and current (ECN) noise-time series of samples exposed to NaCl electrolyte. Figure 8 shows the EPN technique, which presents transitory high-frequency signals, indicating ion reactions. The ECN from Figure 9 shows how AHSS CP 780, Zn_3_(PO_4_)_2_, and Zn_3_(PO_4_)_2_/E-coat presented a transient related to localized corrosion; however, in Table 3 the R_n_ shows how the Zn_3_(PO_4_)_2_ presented a higher resistance to corrosion with 9.048 × 10^6^ Ω·cm^2^, indicating that if behavior is similar, the corrosion resistance is higher. The values in Table 3 indicate that localized corrosion is predominantly present in the system. That indicates that surfaces present heterogeneities. Only ZrO_2_ showed uniform corrosion; however, its corrosion resistance was lower (4.901 × 10^3^ Ω·cm^2^).

The results of the type of corrosion show differences due to the corrosion mechanism. As the coating presented heterogeneity, the attack occurs in preferential zones that are dissolved in a totally different way; for that reason, some results present discordance, but this is due to the mixed corrosion attack.

#### 3.2.3. Electrochemical Impedance Spectroscopy (EIS)

**Figure 10:**
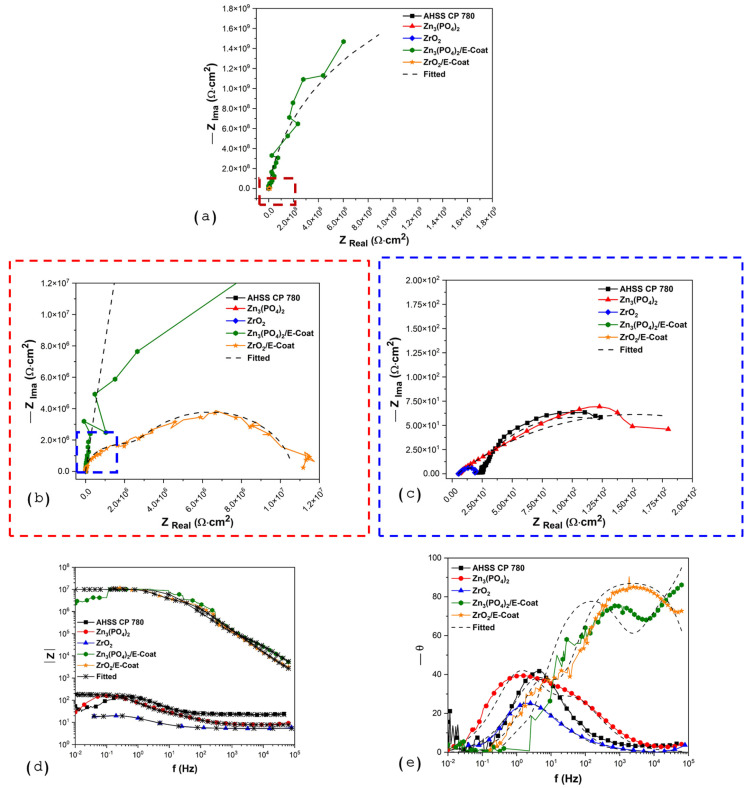
(**a**) Nyquist diagrams, (**b**,**c**) windowing of Nyquist diagrams, and (**d**,**e**) Bode diagrams for CP 780 samples exposed to 3.5 wt. % NaCl solution.

Figure 10 shows the Nyquist and Bode diagrams for pretreatments. The Nyquist diagram from Figure 10a needs the zoom (windowing) shown in Figure 10b,c, where the AHSS CP 780 shows one semicircle, which is supported by the Bode diagrams in Figure 10d,e. Also, the Bode diagram of |Z| shows a low resistance value. The n value (see Table 4) is 0.63, indicating that the surface is heterogeneous and the current is distributed in a non-homogeneous way. Also, Zn_3_(PO_4_)_2_ and ZrO_2_ showed a single time constant; however, the coating is heterogeneous due to the n values of 0.49 and 0.66. The value of R_1_ is 296 Ω·cm^2^, indicating that it is more resistant than ZrO_2_.

On the other hand, the E-coatings exhibited two time constants, indicating that the barrier effect is more effective. The more resistant coating is Zn_3_(PO_4_)_2_/E-coat, and the coatings are more homogeneous because the n value is 0.92. Also, ZrO_2_/E-coat presented an n value of 0.99, indicating that the coating distribution is homogeneous on the surface. The resistance is bigger for Zn_3_(PO_4_)_2_/E-coat with 1.48 × 10^9^ Ω·cm^2^, and ZrO_2_/E-coat just obtained 3.28 × 10^6^. This means an important increase for the corrosion resistance. The values of CPE are in ×10^−9^ F·s^n−1^/cm^2^, indicating that the energy accumulation is low and that the coating helps to reduce ionic transfer.

## 4. Discussion

Electrochemical noise analysis indicates that the absence of a passive layer, combined with metallurgical heterogeneities in the metal matrix, governs the alloy’s corrosion behavior [60,61,69]. Localized corrosion is driven by cathodic and anodic regions formed by phase differences, grain-size variations, contaminants, and microstructural nonuniformity. In these alloys, galvanic corrosion arises from phase disparities [70]. For CP780, the coexistence of multiple phases promotes galvanic coupling: martensite, retained austenite, and ferrite act as cathodes, while bainite serves as an anode. Surface defects further increase susceptibility to localized attack, which, over time, can transition to generalized corrosion through galvanic interactions.

ZrO_2_ coatings have been employed for different alloys and in several applications. Yang et al. [69] mentioned that ZrO_2_ coating modulates the corrosion rate; the corrosion mechanism in that coating is between atoms. Subsequently, the ZrO_2_ film became damaged and even detached, exposing the underlying Mg substrate, which then reacted with the electrolyte. The i_corr_ of the coating is 1.2 × 10^−6^ A/cm^2^ in Peron’s research. In this research, the value was 3.90 × 10^−5^ A/cm^2^, which may be due to the process; however, the e-coating of ZrO_2_ presented a higher resistance with values of ×10^−9^ A/cm^2^, indicating that the coating has better corrosion resistance [71]. The EIS results show that the coatings presented values on the order of ×10^6^ and ×10^5^ for Chang et al. [72]. The results obtained by Chang are within those obtained in this research for ZrO_2_ coatings.

The use of Zn_3_(PO_4_)_2_ coatings has been reported; results from different studies showed that the coating increased corrosion resistance. However, in that research the sample showed pure activation and a large cathodic reaction when evaluated by potentiodynamic polarization [73]. In this research, the cathodic reaction was important due to H+ generation, which can cause brittle coating. The i_corr_ of coated sample presented values of the ×10^−7^ A/cm^2^ order in that research; in this research, it presented values of ×10^−5^ A/cm^2^. The difference is that Yu et al. [71] used dipping in the coating; in this research, the E-coat of Zn_3_(PO_4_)_2_ presented values of ×10^−9^ A/cm^2^, indicating that Zn_3_(PO_4_)_2_ can increase the corrosion resistance when some particles are added to the solution.

**Figure 11:**
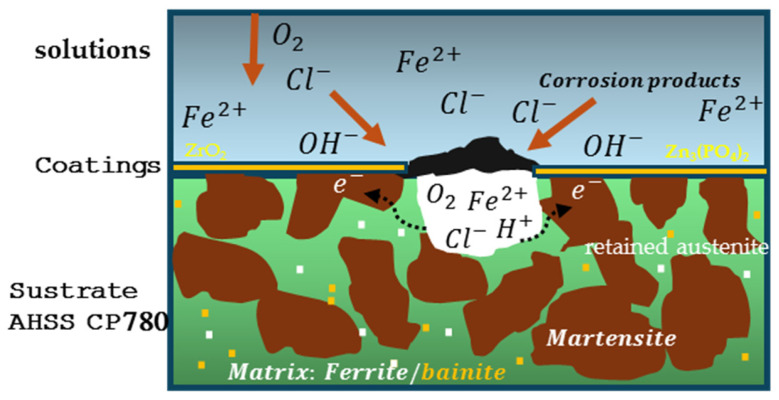
Corrosion mechanisms in coatings of ZrO_2_ and Zn_3_(PO_4_)_2_.

The corrosion mechanism (Figure 11) that was shown in the results obtained by EN is related to mixed systems, as indicated by the relationship between the coating and NaCl solution [74,75,76,77,78]. In this electrolyte, the effect of Cl^−^ attacks is localized; however, H^+^ ions in the cathodic reactions break the coating and make it susceptible to Cl^−^ ion attacks. It is important to mention that the H^+^ role is to begin the cracking of the coating; after that, the Cl^−^ ions attack the coating to dissolve it [79,80,81,82]. The exposed areas of the coating are susceptible to the initiation of corrosion, and with them the substrate phases such as ferrite act as anodic areas of greater dissolution in the corrosion system.

## 5. Conclusions

According to the results of the research, it can be concluded that

- E-coatings achieved 99% and 100% efficiencies due to reduced i_corr_ in coated samples; the conventional pretreatment coatings showed negative protection values. This occurred due to the anodic function of the conventional coating.
- The samples with an E-coat paint coating and pretreatment (Zn_3_(PO_4_)_2_/E-coat and ZrO_2_/E-coat) have the lowest current densities with values of 6.44 × 10^−11^ and 1.02 × 10^−9^ A/cm^2^ and also do not show a tendency towards localized corrosion or negative hysteresis.
- The corrosion mechanism is mixed due to the heterogeneity of the surface of the zinc phosphate and zirconium oxide pretreatments.
- The corrosion resistance of R_n_ is bigger for Zn_3_(PO_4_)_2_/E-coat with 9.048 × 10^6^ Ω·cm^2^, followed by ZrO_2_/coat with 1.84 × 10^5^ Ω·cm^2^. The resistance of AHSS is 1.286 × 10^4^ Ω·cm^2^, which is more than 10 times higher than that of AHSS.
- The EIS results showed that Zn_3_(PO_4_)_2_/E-coat exhibited the highest resistance with 1.48 × 10^9^ Ω·cm^2^. The samples coated with conventional pretreatment methods showed low corrosion resistance values of 296 and 20 Ω·cm^2^.
- The surface of a conventional coating is heterogeneous due to n values in the range of 0.49–0.66. The E-coating showed values of 0.92 and 0.99, indicating that the current distribution is more homogeneous.

## Figures and Tables

**Table 1:** Chemical composition of AHSS CP780 (wt. %) [3].

	AHSS CP780									
**Element**	C	Mn	Ti	P	Cr	S	Si	Nb	Al	Fe
	0.091	1.669	0.007	0.010	0.771	0.002	0.511	0.045	0.034	Balance

**Table 2:** Obtained electrochemical parameters for AHSS CP 780 exposed to 3.5 wt. % NaCl solution.

Samples	E_corr_	i_corr_	E_A-C_	Ba	Bc	Coating Efficiency	Hysteresis
	(V) vs. SCE	(A/cm^2^)	(V) vs. SCE	(mV)	(mV)	(%)	
AHSS CP 780	−0.429	2.41 × 10^−5^	−0.648	63.28	549.42		Positive
Zn_3_(PO_4_)_2_	−0.45	3.48 × 10^−5^	−0.648	80.78	407.67	−44.3	Positive
ZrO_2_	−0.711	3.90 × 10^−5^	−0.638	103.46	215.53	−61.8	Positive
Zn_3_(PO_4_)_2_/E-coat	−0.232	6.44 × 10^−11^	−0.09	775.21	318	100.0	Negative
ZrO_2_/E-coat	−0.444	1.02 × 10^−9^	−0.587	28.6	225.63	99.9	Negative

**Table 3:** Statistics parameters obtained from time-domain analysis.

Samples	R_n_ (Ω·cm^2^)	LI	Corrosion Type	Kurtosis	Corrosion Type	Skew	Corrosion Type
AHSS CP 780	1.286 × 10^4^	0.10	Mixed	14.83	Localized	3.00	Localized
Zn_3_(PO_4_)_2_	1.212 × 10^4^	0.10	Mixed	29.00	Localized	4.75	Localized
ZrO_2_	4.901 × 10^3^	0.0058	Uniform	6.30	Localized	0.04	Uniform
Zn_3_(PO_4_)_2_/E-coat	9.048 × 10^6^	0.99	Localized	1.62	Uniform	0.03	Uniform
ZrO_2_/E-coat	1.84 × 10^5^	0.12	Localized	8.33	Localized	1.51	Localized

**Table 4:** Electrochemical characteristics from Nyquist diagrams of AHSS CP 780 samples exposed to 3.5 wt. % NaCl solution.

Sample	R_sol_ (Ω·cm)	CPE_1_ (μF/cm^2^)	n_1_	R_1_ (Ω·cm)	CPE_2_ (μF/cm^2^)	n_2_	R_2_ (Ω·cm)	Error	X^2^
AHSS CP 780	9.63	1.70 × 10^−3^	0.63	2896	---	---	---	<3.48	1 × 10^−2^
Zn_3_(PO_4_)_2_	5.13	2.80 × 10^−3^	0.49	296	---	---	---	<2.5	2 × 10^−3^
ZrO_2_	5.31	1.76 × 10^−2^	0.66	20.73	0.112	0.72	6.843	<4.30	6 × 10^−3^
Zn_3_(PO_4_)_2_/E-coat	153.5	1.42 × 10^−9^	0.92	1.48 × 10^9^	---	---	---	<3.2	1 × 10^−3^
ZrO_2_/E-coat	122.5	1.17 × 10^−9^	0.99	3.28 × 10^6^	1.17 × 10^−8^	0.922	7.36 × 10^6^	<5.5	7 × 10^−2^

## Data Availability

The original contributions presented in this study are included in the article. Further inquiries can be directed to the corresponding authors.
